# Reasons for non-disclosure of HIV-Positive status to healthcare providers: a mixed methods study in Mozambique

**DOI:** 10.1186/s12913-023-09865-y

**Published:** 2023-08-30

**Authors:** Laura Fuente-Soro, Antía Figueroa-Romero, Sheila Fernández-Luis, Orvalho Augusto, Elisa López-Varela, Edson Bernardo, Anna Saura-Lázaro, Paula Vaz, Stanley C. Wei, Peter R. Kerndt, Tacilta Nhampossa, Denise Naniche

**Affiliations:** 1 ISGlobal, Hospital Clínic - Universitat de Barcelona, Barcelona, Spain; 2 Centro de Investigação Em Saúde de Manhiça (CISM), Maputo, Mozambique; 3 Fundação Ariel Glazer Contra O SIDA Pediatrico, Maputo, Mozambique; 4 U.S Centers for Disease Control and Prevention (CDC), Maputo, Mozambique; 5 Instituto Nacional de Saúde (INS), Maputo, Mozambique

**Keywords:** Awareness, HIV disclosure, Barriers, HIV-testing, Mozambique, Sub-Saharan Africa

## Abstract

**Background:**

Non-disclosure of known HIV status by people living with HIV but undergoing HIV testing leads to waste of HIV testing resources and distortion of estimates of HIV indicators. In Mozambique, an estimated one-third of persons who tested positive already knew their HIV-positive status. To our knowledge, this study is the first to assess the factors that prevent people living with HIV (PLHIV) from disclosing their HIV-positive status to healthcare providers during a provider-initiated counseling and testing (PICT) campaign.

**Methods:**

This analysis was nested in a larger PICT cross-sectional study performed in the Manhiça District, Southern Mozambique from January to July 2019, in which healthcare providers actively asked patients about their HIV-status. Patients who tested positive for HIV were crosschecked with the hospital database to identify those who had previously tested positive and were currently or previously enrolled in care. PLHIV who did not disclose their HIV-positive status were invited to participate and provide consent, and were interviewed using a questionnaire designed to explore barriers, patterns of community/family disclosure, and stigma and discrimination.

**Results:**

We found that 16.1% of participants who tested positive during a PICT session already knew their HIV-positive status but did not disclose it to the healthcare provider. All the participants reported previous mistreatment by general healthcare providers as a reason for nondisclosure during PICT. Other reasons included the desire to know if they were cured (33.3%) or to re-engage in care (23.5%). Among respondents, 83.9% reported having disclosed their HIV-status within their close community, 48.1% reported being victims of verbal or physical discrimination following their HIV diagnosis, and 46.7% reported that their HIV status affected their daily activities.

**Conclusion:**

Previous mistreatment by healthcare workers was the main barrier to disclosing HIV-positive status. The high proportion of those disclosing their HIV status to their community but not to healthcare providers suggests that challenges with patient-provider relationships affect this care behavior rather than social stigma and discrimination. Improving patient-provider relationships could increase trust in healthcare providers, reduce non-disclosures, and help optimize resources and provide accurate estimates of the UNAIDS first 95 goal.

## Background

To end the HIV epidemic by 2030, in 2014, the Joint United Nations Program on HIV/AIDS (UNAIDS) set the 95–95-95 targets establishing that, by 2030, 95% of all people living with HIV (PLHIV) should be diagnosed, 95% of all them should receive antiretroviral therapy (ART), and 95% of those receiving antiretroviral treatment (ART) should be virally suppressed. Thus, if these targets are achieved, at least 86% of all PLHIV will be virally suppressed and therefore will be unable to transmit HIV [1, 2]. In 2019 there were 1.7 million people newly infected with HIV and 38.0 million PLHIV worldwide [3]. Mozambique had 2,200,000 PLHIV [4], with an HIV prevalence among the adult population (aged 15 to 49 years) of 12.6% in 2019 [4]. However, according to UNAIDS, in 2019, only 77% of PLHIV in Mozambique knew their status, 59% were on treatment and 45% were virally suppressed [4]. In the Manhiça District of Southern Mozambique, where the community HIV-prevalence in 2015 was 33.6%, 75.9% of men and 88.9% of women were aware of their status [5].

HIV t step to entering the HIV cascatesting and counseling (HTC) is the firsde of care and receiving appropriate ART. Provider-initiated counseling and testing (PICT) was implemented to scale up HIV testing at health facility level and to achieve the first UNAIDS 95 goal by offering HTC to all individuals seeking care. However, a previous study conducted in 2015 in Southern Mozambique quantified, for the first time worldwide, that during PICT campaigns almost one third of individuals testing HIV-positive were already diagnosed and enrolled in care [6]. Non-disclosure of a previous HIV-positive diagnosis to the healthcare provider leads to repeat HIV testing among individuals already diagnosed, distorting the number of people that start care in an already overburdened healthcare system. In addition, this distorts programmatic indicators by underestimating the true proportion of PLHIV who are aware of their status and overestimating the number of individuals newly HIV-diagnosed [6]. Although reasons for non-disclosure in the family context have been previously described and associated with stigma and poor retention in HIV care and treatment [7–10], no studies have explored reasons for non-disclosure to the healthcare provider in the context of HIV-testing. Understanding the difficulties faced by PLHIV in disclosing their previous diagnosis to healthcare providers will aid in improving their re-engagement and retention in HIV care, in optimizing resources and in obtaining more accurate estimates of the 95–95-95 targets in high HIV-burden areas. This study sought to explore, factors that prevent PLHIV from disclosing their previous HIV diagnosis during a PICT session in four rural health facilities in the Manhiça district, Southern Mozambique.

## Methods

### Study area and population

This study was performed among youths (> 15 years old) and adults attending primary care in four health facilities in the Manhiça District (Manhiça District Hospital (MDH), Xinavane Rural Hospital and Palmeira and Maragra Peripheral Health Units) from January to July 2019. This region is a semi-rural area located in Maputo province, Southern Mozambique, with an overall community HIV-prevalence of 36.6% in 2015 [5], where the Centro de Investigação em Saúde de Manhiça (CISM) has conducted a continuous health and demographic surveillance system (HDSS) for vital events since 1996. The HDSS registers births, deaths, migrations, pregnancies and household characteristics [11].

At each health facility, HIV-services are delivered free of charge, including HIV-testing [11]. Provider-initiated HIV counseling and testing (PICT) was the most widespread testing modality in the district whereby the provider recommends HTC as part of a primary clinical evaluation according to patient’s symptoms and HIV risk factors.

Routine patient‐level HIV clinical data are recorded on paper since the day of HIV diagnosis and transcribed daily into an electronic Patient Tracking System (ePTS) which is co-managed by the Ministry of Health and other stakeholders.

### Study design and procedures

This is a cross-sectional analysis nested in a larger study which aimed to evaluate the efficiency of a specific provider training proposed by the Mozambican Ministry of Health (MoH) to increase the yield of new diagnoses through targeted PICT. Initially, healthcare providers performed the primary clinical consultation and recommended HIV-testing according to national guidelines for suggestive symptoms, or HIV risk behaviors [12]. Referred patients then visited an HIV counselor, who probed them again about their HIV status. For study procedures, during the counseling session, a study questionnaire exploring socio-demographic characteristics, HIV-related symptoms and sensitive HIV risk behaviors was completed for each participant. To evaluate any impact in the diagnostic yield, universal testing was performed for all individuals that self-reported a previous HIV-negative result or those with unknown HIV status, independently of the symptoms and risk factors. Individuals who self-reported a previous HIV diagnosis were not tested. The identity of all participants who tested HIV-positive during the campaign was cross-checked with the ePTS to detect participants previously diagnosed and enrolled in HIV care but who did not disclose their HIV-positive status to the health provider.

For the purposes of this analysis, we only included those participants who reported being HIV-negative to the healthcare provider or with unknown HIV status but were identified through the ePTS system as previously diagnosed and enrolled in HIV care and the following definitions were used:

- Awareness: refer to all known PLHIV who self-reported a previous HIV-positive diagnosis or who are registered in the ePTS.
- Non-disclosure participants refer to PLHIV who did not disclose their previous HIV-positive diagnosis to the healthcare provider during targeted PICT and were then identified through the ePTS. Two groups were created based on the timing and manner of identification.
- Partial non-disclosures: individuals who disclosed their HIV-status to the counselor during the pre-counseling session before undergoing HIV-testing.
- Complete non-disclosures: individuals who did not disclose their HIV-status to either the provider or the counselor and underwent HIV-testing.
- External stigma was defined as negative attitudes and behaviors that others direct towards PLHIV, which can result in their devaluation or the perception of having less dignity and value than others [13].
- Internal stigma refers to negative attitudes and feelings that individuals may have towards themselves due to their stigmatized identity, such as feelings of shame [13].
- Discrimination was defined as treating someone in a different, unfair or detrimental way, because of his/her HIV condition [13].

### Data collection and data management

Data was captured during the study visit in two electronic questionnaires directly uploaded into REDCap database (Research Electronic Data Capture) [14]. The first questionnaire was completed for all individuals participating in the larger study, including sociodemographic data, HIV risk behaviors, and clinical symptoms, while the second questionnaire, specifically designed for this sub-study, was conducted only among PLHIV who do not disclose their HIV status. This questionnaire explored barriers to disclosing the HIV-positive status to the healthcare provider/counselor, patterns of disclosure amongst acquaintances and family, and questions about stigma and discrimination, extracted from “The People Living with HIV Stigma Index survey” [13].

### Qualitative and quantitative data analysis

Semi-structured interviews were conducted to explore factors that prevent PLHIV from disclosing their status to healthcare providers. The interviews utilized a pre-defined codebook of potential factors based on previous literature on linkage and retention in care [6, 15–17], as well as an open text field for respondents to elaborate on their answers if necessary [18]. Responses from the open text field were then coded and tabulated along with the other predefined codes in an Excel matrix and triangulated by three experienced HIV researchers. Similar barriers were grouped in conceptual categories, and any discrepancies were resolved through discussions and reclassification of codes when necessary. The cumulative frequency of reporting for each barrier and category was assessed, and the number of participants that reported each category was calculated. As participants could report multiple barriers the number of participants referring to a specific category (not individual barrier) was counted only once per category, even if they reported more than one barrier from the same category.

A descriptive analysis was conducted to examine the baseline characteristics of the participants, stratified by the type of disclosure. This study investigated the relationship between disclosure status and several factors, such as patterns of disclosure among close community members, HIV suggestive symptoms, risk behaviors, belonging to HIV key populations, and other variables used to describe the perception of external and internal stigma and discrimination. The statistical analyses used to assess baseline characteristics and the disclosure status of the participants were the chi-square and Fisher's exact tests, together with the corresponding 95% confident interval (95% CI).

## Results

### Study profile

**Fig. 1:**
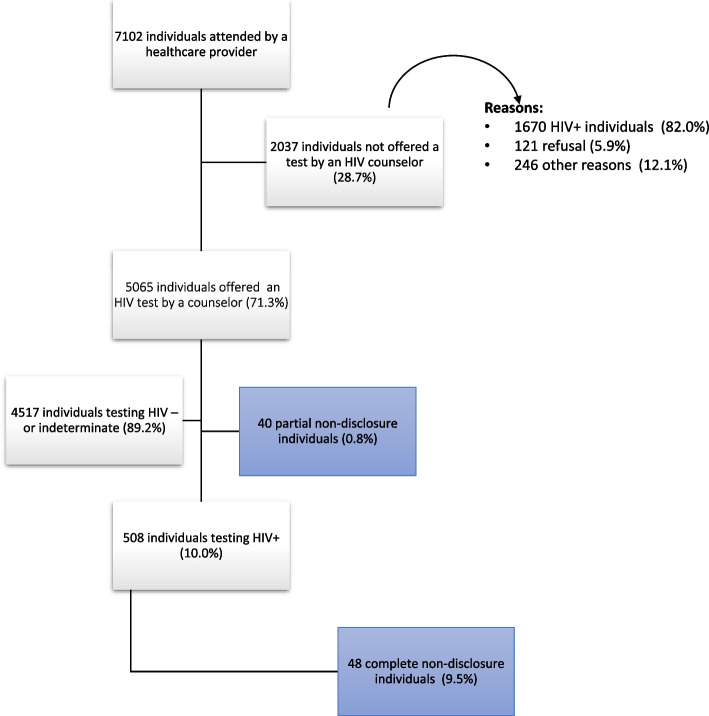
Study profile

Overall, in the four health facilities of the Manhiça District, 7102 adults reporting to the primary or emergency care settings consented to participate in the larger study and were assessed for HIV testing criteria following PICT procedures (Fig. 1). Among those, 71.3% (5065/7102) (95% CI 70.3–72.4) were offered an HIV test by a counsellor while 28.7% (2037/7102) (95% CI 27.6–29.7) were not referred for testing mainly because they self-reported a previous HIV-positive diagnosis (23.5%, 1670/7102). After the first screening performed by the health provider, during the counseling and testing session, 40 individuals (0.8%, 95% CI 0.6–1.1) disclosed their positive HIV status to the counselor just prior to the test (Partial non-disclosure), and thus were not tested. Out of the 508 individuals who tested HIV-positive during the study, 48 (9.4%,95% CI 7.0–12.3) were identified as PLHIV who did not disclose their status through the ePTS.

Finally, of the 88 participants who did not disclose their status, 81 (92.0%) agreed to respond to the specific questionnaire design for the purpose of this sub-analysis.

### Baseline characteristics among PLHIV who do not disclose their HIV status

Among the 81 study participants, two-thirds (66.7%) were women, and mean age was 39.4 years (IQR 28.0 -48.0) (Table 1). Differences were not found by type of non-disclosure. In the MDH and the secondary health facilities the proportion of individuals that do not disclose their HIV status at all (complete non-disclosures) was higher compared with those individuals that disclosed their status prior to undergoing testing (partial non-disclosures) (86.2% and 67.3% respectively), whereas in the rural Hospital (Xinavane) partial non-disclosures predominated (66.7%).

**Table 1:** Baseline characteristics of the participants (*n* = 81) identified during the PICT campaign according to type of non-disclosure either partial or complete

		**Partial non-disclosure (*n* = 33)**	**Complete non-disclosure (*n* = 48)**	**Total** **(*n* = 81)**	p**
		N (%)	N (%)	N	
Gender					
	Men	10 (37.0%)	17 (63.0%)	27	0.631
	Women	23 (45.6%)	31 (57.4%)	54	
Age (years)					
	18–24	6 (42.9%)	8 (57.1%)	14	0.288
	25–34	11 (55.0%)	9 (45.0%)	20	
	35–44	5 (25.0%)	15 (75.0%)	20	
	≥ 45	11 (40.7%)	16 (59.3%)	27	
Marital status					
	Married/in a relationship	20 (42.6%)	27 (57.4%)	47	0.536
	Divorced/separated/widowed	8 (47.1%)	9 (52.9%)	17	
	Single	5 (29.4%)	12 (70.6%)	17	
Occupation^a^					
	Agriculture sector	10 (34.5%)	19 (65.5%)	29	0.650
	Service sector	7 (50.0%)	7 (50.0%)	14	
	Construction	2 (50.0%)	2 (50.0%)	4	
	Student/Unemployed	14 (48.3%)	15 (51.7%)	29	
Partner occupation^a^					
	Agriculture sector	6 (54.5%)	5 (45.5%)	11	0.707
	Service sector	4 (50.0%)	4 (50.0%)	8	
	Construction	2 (28.6%)	5 (71.4%)	7	
	Student/Unemployed	8 (53.3%)	7 (46.7%)	15	
	Do not have a partner	15 (38.2%)	21 (61.8%)	36	
Health unit					
	Manhiça District Hospital	4 (13.8%)	25 (86.2%)	29	< 0.001
	Xinavane Rural Hospital	24 (66.7%)	12 (33.3%)	36	
	Palmeira and Maragra Health Peripheral Centers	5 (31.3%)	11 (68.7%)	16	

^a^for complete non-disclosures, numbers included in each sub-analysis are not be equal due to missing data**p-value was calculated using chi-square or Fisher’s exact test

### Disclosure of HIV-status and implications for the first UNAIDS target

Overall, in this cohort, there were 2218 individuals living with HIV (including 1670 participants that self-reported their HIV status, 40 participants that report their status prior to HIV testing, and 508 individuals that tested positive). Of the 548 PLHIV who should have been referred for HIV testing as part of the provider-initiated testing and counseling strategy in ideal conditions, 16.1% (88/548, 95% CI 13.1–19.4) were previously diagnosed and enrolled in HIV care.

To assess progress towards the first UNAIDS 95 target, we used the definition of awareness of HIV status as a proxy. Our results showed that 79.3% (1758/2218, 95% CI 77.5–80.9) of the PLHIV in this cohort were already aware of their HIV-positive status. However, under routine care conditions, where the participants would not have been asked subsequently by the provider and counselor about their HIV status, the 88 individuals who did not disclose their HIV status would have been considered newly diagnosed with HIV. This would have resulted in an underestimate of the first UNAIDS 95 by 4%. Therefore, the estimated proportion of participants aware of their HIV-positive status would have been 75.3% (1670/2218, 95% CI 73.4–77.1) and the true proportion of individuals newly diagnosed would be overestimated from 20.7% (95% CI 19.5–22.5) to 24.7% (95% CI 22.9–26.6).

### Social behavior among PLHIV who do not disclose their HIV status

Regarding HIV-related risk behaviors, 49.2% (29/59) of the respondents reported never using condoms during sexual relationships and 8.6% (7/81) reported having a partner living with HIV (Table 2). Additionally, we explored patterns of disclosure of HIV-status to their community, and most of the participants (68/81, 83.9%) reported that they had talked to someone about their HIV-status since their first HIV diagnosis. Almost half of them (39/81, 48.2%) disclosed their HIV-status to someone on the day of their diagnosis. Overall, among participants with a partner (*n* = 49), 77.5% (38/49) stated that their partner knew their HIV-status, and 63.2% (31/49) reported that they talked about their diagnosis with them. Regarding reported feelings during the disclosure process, only 42.0% (34/81) reported having felt understood or supported when disclosing their condition, while 17.3% (14/81) mentioned that the other person felt disappointed and 9.9% (8/81) surprised (Table 2).

**Table 2:** Clinical characteristics, risk behaviors and patterns of disclosure to close community (*n* = 81)

Suggestive symptoms of HIV*	N	%
**Any suggestive symptoms of HIV**^a^	**13**	16.1%
Prolonged or recurrent respiratory distress	1	7.7%
Genital discharge or genital wounds	1	7.7%
Diarrhea	1	7.7%
Fever of fever syndrome for more than three weeks	2	15.4%
Skin itching	1	7.7%
Other	8	6.2%
**HIV-related risk behaviors**		
**Hospitalization during the past 6 months**	4	4.9%
**Current sexual partner living with HIV**	7	8.6%
**More than one sexual partner during the past year**	5	6.2%
**Did not use condom in any of previous sexual relations (*n* = 59)**	29	49.2%
**Occasionally use of condom in previous sexual relations (less than half of the times) (*n* = 59)**	14	23.7%
**Other vulnerable groups**		
**Pregnant woman (*n* = 54)**	1	1.9%
**Partner of a pregnant woman (*n* = 27)**	1	3.7%
**Patterns of disclosure to close community**		
**Disclosure of the HIV-status to someone in the community**		
Yes	68	83.9%
No	11	13.6%
NI	2	2.5%
**Relationship with the person you usually talk to about your HIV-status (multiple answer)**		
Partner	**31**	**38.7%**
Parents	28	34.6%
Children	8	9.9%
Other relatives	6	7.4%
Non-relative individuals	9	11.1%
No one	11	13.6%
NI	3	3.7%
**Disclosure of the HIV status to the people cohabiting in the household**		
Yes, all of them	19	23.5%
Yes, some of them	30	37.0%
No	24	29.6%
Lives on his/her own	7	8.6%
Does not know	1	1.2%
**Relationship with the person cohabiting who knows about the HIV status (multiple answer) (*n* = 49)**		
Partner	38	77.5%
Parents	20	40.8%
Children	13	26.5%
Other relatives	6	12.2%
Lives on his/her own	7	14.3%
NI	2	4.1%
**Reaction of the first person who knew about your HIV status in the community (multiple answer)**		
Disappointed	14	17.3%
Supportive/ Understanding	34	42.0%
Happy	1	1.2%
Scared	1	1.2%
Refusal/ Angry	4	4.9%
Surprised	8	9.9%
No reaction	1	1.2%
**NI**	**26**	**28.4%**

^a^Reporting suggestive symptoms of HIV in the last 12 months was defined as reporting headache, diarrhea, wounds, rash, skin itching, genital wounds, coughs for more than 3 weeks, night sweats, sore throat, fever, cardiac palpitations, dizziness, weight loss, insomnia, memory loss, loss of appetite, joint pain, abdominal pain, numbness or tingling sensation in arms and legs, respiratory distress, pain when urinating, swollen lymph nodes, white spots in mouth [12]

### Factors related to non-disclosure of HIV-positive status to the healthcare provider

Out of the 81 respondents, a total of 185 self-reported factors were identified that prevented disclosure of HIV-positive status, which were then grouped into 7 conceptually similar categories. Table 3 summarizes the frequency of reporting of these factors based on the number of participants that reported them.

**Table 3:** Barriers to disclosure of HIV-positive status to healthcare providers

Why did you decide not to disclose your HIV positive status to the healthcare provider and repeat the test?	(A) Frequency of reporting (n, %)	(B) Number of individuals that reported the barrier (n, %)
**Mistreatment by the healthcare providers**	**81 (43.8%)**	**81 (100%)**
I was mistreated by the healthcare provider	81 (100%)	81 (100%)
**Willingness to reengage in care**	**29 (15.7%)**	**29 (35.8%)**
I changed my address	4 (13.8%)	4 (4.9%)
I want to initiate the treatment in this health unit	2 (6.9%)	2 (2.5%)
I want to re-initiate the treatment	6 (20.7%)	6 (7.4%)
It is the only way I know to reinitiate the treatment	4 (13.8%)	4 (4.9%)
It has been some time since I last came to this health unit	6 (20.7%)	6 (7.4%)
I stopped the treatment	7 (24.1%)	7 (8.6%)
**Desire to see if been cured**	**27 (14.6%)**	**27 (33.3%)**
I wanted to confirm that I was cured	27 (100%)	27 (33.3%)
**Healthcare provider miscommunication**	**25 (13.5%)**	**25 (30.9%)**
I did not understand that I had to repeat the test when I signed the informed consent	7 (28.0%)	7 (8.6%)
The provider did not ask me about my HIV status	18 (72.0%)	18 (22.2%)
**Distrust in the former positive result**	**13 (7.0%)**	**13 (16.0%)**
I did not believe the former HIV-positive result	13 (100%)	13 (16.0%)
**Stigma and discrimination**	**6 (3.2%)**	**6 (7.4%)**
Nobody knows me here	1 (16.6%)	1 (1.2%)
I do not want anyone to know that I am HIV positive	2 (33.3%)	2 (2.5%)
I was afraid of being scolded	1 (16.6%)	1 (1.2%)
Family refusal	1 (16.6%)	1 (1.2%)
Shame	1 (16.6%)	1 (1.2%)
**Other**	**4 (2.2%)**	**4 (4.9%)**
I never had a positive result before	2 (50.0%)	2 (2.5%)
I do not want to respond	2 (50.0%)	2 (2.5%)

Categories of barriers are shown in bold letters and individual barriers are shown below each category

For each category of barrier refers to the number of participants that reported at least one of the barriers included in the category among the total of participants (*N* = 81). Percentages are calculated A) among the total of self-reported barriers (*n* = 185) and B) among the total of individuals (*N* = 81)

All participants reported previous mistreatment by healthcare providers. In addition to mistreatment, other frequently reported reasons for not disclosing HIV-positive status included the desire to see if they were cured (33.3%, 27/81), miscommunication challenges between the healthcare provider and the participant (30.9%, 25/81), or desire to reengage in care (23.5%, 19/81). Several participants expressed that re-testing is the only way to reengage HIV care in a new health facility. Moreover, distrust in the previous positive test result was also reported as a factor that prevented disclosure (16.0%, 13/81). Only a small number of participants (7.4%, 6/81) expressed factors directly related to stigma and discrimination.

### Stigma and discrimination

While stigma and discrimination were not among the main self-reported barriers to disclosing HIV-positive status to the healthcare provider, Table 4 shows that when participants were specifically asked about the impact of their HIV status in their daily life, almost half of them (44.4%, 36/81) reported that they had stopped doing some activities. Of these, 18.5% (15/81) had stopped going to the clinic and 7.4% (6/81) reported that they did not go to the hospital when they needed to. Furthermore, 42.0% (34/81) of the participants reported experiencing negative feelings related to their HIV status over the past year, while 48.1% (39/81) reported having been victims of gossip, insults or physical attacks that they attributed to their HIV status. A quarter of the participants (21/81, 25.9%) reported hearing gossip about themselves, particularly women (35.2% compared to 7.4% for men, *p* = 0.006), and 9.9% (8/81) reported being physically attacked at least once during the previous 12 months.

**Table 4:** Stigma and discrimination suffered by people living with HIV who do not disclose their status among their close community

**Experiences of stigma and discrimination**	N	%
**Has seen anyone gossiping about her/him**		
Yes	21	25.9%
No	54	66.7%
NI	6	7.4%
**Frequency of insults or threats during the past 12 months**		
Never	71	87.7%
Once	6	7.4%
Occasionally	4	4.9%
**Frequency of physical attacks during the past 12 months**		
Never	73	90.1%
Once	5	6.2%
Occasionally	3	3.7%
**Internal stigma**	N	%
**Feelings resulting from HIV-condition during the past 12 months (multiple answer)**		
Ashamed	17	21.0%
Guilty	5	6.2%
Blamed others	2	2.5%
With low self-esteem	13	16.0%
Should be punished	1	1.2%
Wants to hurt her/himself	1	1.2%
Furious	2	2.5%
Scared	1	1.2%
Fine	47	58.0%
NI	2	2.5%
**Stopped doing any of the following items as a result of HIV-condition during the past 12 months**		
Stopped going to the clinic	15	18.5%
Stopped going to school	0	0%
Stopped having sex	1	1.2%
Stopped looking for a job/a promotion	0	0%
Decided not to get married	1	1.2%
Stopped working	1	1.2%
Isolated from family and friends	6	7.4%
Stopped participating in social events	4	4.9%
Decided not to have more children	2	2.5%
Did not go to the hospital when he/she had to	6	7.4%
Nothing changed	43	53.3%
NI	5	6.2%

*NI* No information

*N* for each barrier refers to the number of participants that reported the barrier and percentages are calculated among the total of individuals (*N* = 81)

## Discussion

To our knowledge, this is the first study that explored factors that prevent PLHIV from disclosing their previous HIV-positive status to healthcare providers in the context of HIV-testing campaigns in southern Mozambique. We found that, among individuals referred for HIV-testing by the healthcare provider and identified as a person living with HIV by an HIV counselor, about one fifth were previously diagnosed with HIV and enrolled in care but did not disclose their previous status to the healthcare provider. Mistreatment by the healthcare provider was the most reported factor that prevented disclosing in the context of PICT and was expressed by all participants. Moreover, the desire to re-engage in care after being lost to follow up was a commonly reported reason to not disclose and re-test. Other factors included the desire to see if they have been cured, and a lack of efficient communication between the healthcare provider and the individual. Nevertheless, among those non-disclosing individuals, a high proportion (83.9%) reported having disclosed it within their close community. Almost half of the participants (46.9%) stopped doing some routine activities due to their HIV-positive status and 48.1% declared themselves as having been victims of gossip, insults or physical attacks during the previous year due to their serostatus.

Our findings suggest that one in six people referred for HIV-testing in a PICT context were already diagnosed. This proportion of individuals who did not disclose their HIV-status to a healthcare provider has remained high in the context of PICT during the past five years. In 2015, in a similar study conducted in Manhiça, the proportion of non-disclosures detected during a PICT campaign was 29.4% [5]. Although our findings suggested a lower proportion of non-disclosure (16.1%) this reduction may be due to proactive efforts by providers and counselors to reduce the expected proportion of non-disclosure patients and to the improvement in the PICT strategy over the years. Changes in HIV-testing policies, such as the implementation of Universal Treatment in Mozambique that began in mid-2016 [19], may have had an impact on the reduction of the number of PLHIV who do not disclose their HIV status by expanding the access to HIV diagnosis and care, improving healthcare providers’ skills in identifying and counseling PLHIV, and thus increasing the potential willingness among individuals to disclose their HIV-positive status.

In our study we found that all PLHIV who do not disclose their HIV status had previously experienced situations of mistreatment from health care providers and referenced those situations as a barrier to do not disclose their status to the clinician. Previous studies demonstrated that disrespectful attitudes towards PLHIV in the context of clinical care are also key barriers to follow up.

In some cases, mistreatment by healthcare providers, including negative attitudes and practices towards PLHIV, may result from factors such as burn-out syndrome among healthcare workers [20], which has been widely reported in sub-Saharan Africa [21, 22], or misunderstanding stemming from ineffective communication between healthcare providers and PLHIV who may have received a previous diagnosis but did not disclose their status. Lack of health literacy leads to unsafe health practices [23, 24] and healthcare providers should be able to understand the context of the individual that influences how healthcare information is received.

Patient-centered approaches may be useful to improve communication between healthcare providers and PLHIV. In this sense, a study performed in Tanzania in 2017 to improve the ability of healthcare providers to identify non-adherent patients by identifying problems of communication with patients, increased the percentage of patients reporting to health providers their non-adherence from 3.3% to 10.7% in two months [25]. In addition, peer-based educational interventions may be beneficial to increase HIV literacy [26]. A meta-analysis performed in 2018 that included studies performed in developing countries found that peer education interventions appeared to be effective in informing individuals about HIV transmission routes [27]. Additionally, we found that non-disclosure of HIV-positive status as a means to re-engage in care represented 15.7% of the barriers cited by participants. Thus, although we did not investigate previous retention in care, there is likely to be overlap between the non-disclosing re-testers and those who dropped out of care.

The study found that among participants who did not disclose their HIV status to healthcare providers, the majority of them (84.0%) had disclosed it to their close community, indicating that these two disclosure processes face different challenges. Extensive efforts to identify and address barriers to HIV disclosure to sexual partners, such as shown through previous research [8, 10, 28–30], could explain these high rates of disclosure. However, there is a lack of research on interventions aimed at reducing the number of PLHIV who do not disclose their status to healthcare providers.

In our study, stigma and discrimination only represent 3.2% of reported barriers. However, 48.1% of the participants reported experiencing gossip, threats or attacks in the past year, a lower proportion than the 56% found in the 2013 Mozambican National Stigma Index Survey [31]. These results suggest that while efforts to reduce HIV-related stigma in Mozambique may have had some success, stigma and discrimination continue to be prevalent at the community level. One possible explanation for this decrease in stigma may be the 2014 law passed by the Mozambican government that aimed to protect the rights and dignity of PLHIV [32]. However, it is important to note that PLHIV who do not disclose their HIV status to health providers in this study may have unique characteristics and perceptions of stigma that differ from those in the PLHIV population surveyed in the Mozambican National Stigma Survey [31], that included PLHIV in general. As such, comparisons between the two populations should be made with caution.

Finally, in the absence of a mechanism to identify non-disclosures, the estimated proportion of individuals aware of their HIV status would be 75.3% (95% CI 73.4–77.1), while after their identification, this proportion rises to 79.3% (95% CI 77.5–80.9), closer to the 95% goal. The most recent AIDS impact survey in Mozambique found that 16% of PLHIV with blood evidence of antiretroviral therapy and/or viral suppression reported that they had never been tested for HIV, and 22% reported a negative result from their last HIV test [33]. In this context, the practice of reclassifying such individuals as knowing their status for estimation of the first 95% goal is important.

This study has several limitations. First, each health facility has its own electronic Patient Tracking System database, thus individuals presenting at a facility for testing but enrolled in HIV care in other National Health Facilities will be considered new diagnoses. In addition, patients may have received a previous positive test result but not linked to care, in which case they would not appear in the ePTS system, and thus estimates of non-disclosures could be underestimated. Second, the questionnaires used for this study were designed considering previous literature and the results of former studies performed in the same region of Mozambique [6, 15]. Thus, despite the open-answer nature of the questionnaire, the design of the questionnaire may have focused the results towards the predefined categories, thus some potential barriers for disclosing HIV-positive status to the healthcare provider may have been omitted or underestimated. Finally, for each category of barriers, we attempt to assess the association between that specific barrier to do not disclose and the patient characteristics through a bivariate and multivariate analysis, however, due to the relatively small sample size the model was underpowered. Thus, the interpretation of reported associations should be interpreted with caution and further research should be conducted to consolidate such associations and potentially confusing factors.

## Conclusions

In conclusion, our study identified mistreatment by healthcare providers as the most reported factor that prevented PLHIV from disclosing their previous HIV-positive status to healthcare providers in the context of PICT campaigns. Patient-centered approaches and peer-based educational interventions may be useful to improve communication between healthcare providers and PLHIV, ultimately increasing the potential willingness among individuals to disclose their HIV-positive status. The high proportion of non-disclosing individuals who disclosed their status within their close community highlights the importance of continuing to address community-level stigma and discrimination. Our findings underscore the need for efforts to reduce mistreatment by healthcare providers and address community-level stigma and discrimination to increase disclosure of HIV-positive status to healthcare providers, ultimately improving access to HIV care and treatment. Finally, by reducing the number of non-disclosures, scarce resources will not be wasted on re-testing individuals that have previously been diagnosed with HIV, and critical programmatic indicators will not be underestimated, leading to more accurate estimates of the UNAIDS first 95 goal.

## Data Availability

There are ethical restrictions on sharing a de-identified data. Data contain potentially sensitive information and national ethics committee in Mozambique does not authorize data sharing without a data sharing request specifying the objectives and the researchers who will have access to the data.

## Abbreviations

UNAIDS: Joint United Nations Program on HIV/AIDS
PLHIV: People living with HIV
ART: Antiretroviral therapy
HTC: HIV-testing and counseling
PICT: Provider-initiated counseling and testing
MDH: Manhiça District Hospital
CISM: Centro de Investigação em Saúde de Manhiça
HDSS: Health and demographic surveillance system
ePTS: Electronic Patient Tracking System
CDC: Centers for Disease Control and Prevention
CI: Confidence interval
